# Post-competition psychological recovery in elite adolescent athletes: a technology-scaffolded perspective

**DOI:** 10.3389/fpsyg.2026.1902066

**Published:** 2026-08-13

**Authors:** Junwei Zeng, Yan Zhang, Nuan Wen, Shan He

**Affiliations:** 1 School of Physical Education, Guangdong University of Technology, Guangzhou, China; 2 School of Education, City University of Macau, Taipa, Macao SAR, China

**Keywords:** conversational AI, digital intervention, emotion regulation, psychological recovery, technological scaffolding, virtual reality

## Abstract

In tightly scheduled competitions, elite adolescent athletes often face compressed recovery windows while their self-regulatory capacities remain under development. This developmental mismatch creates a practical regulation gap in the hours following competition, as heightened emotional activation delays downregulation and psychological recovery, thereby affecting subsequent performance. To address this challenge, the current perspective suggests that digital technologies can serve as external scaffolds to facilitate athletes’ psychological recovery during critical post-competition windows. We propose a technology-scaffolded regulation framework that conceptualizes recovery as a hierarchical process progressing from physiological anchoring to emotional reframing and cognitive recharging. Within the framework, virtual reality, conversational AI, and related technologies are considered potential supports that may reduce cognitive burden and extend recovery assistance when self-regulation is most fragile. This framework establishes a foundation for future empirical research on technology-supported psychological regulation and offers practitioners a structured pathway to deliver precise support during critical post-competition recovery windows.

## 1 Introduction

Although participation in sport can foster enjoyment, competence, and personal growth, the pathway to elite performance during adolescence is also marked by sustained and often cumulative demands (Alfermann and Stambulova, 2007). To cultivate a high-level athletic career, young athletes must invest considerable effort from an early age, accumulating the technical, tactical, and competitive experiences required to progress toward senior elite sport and, for some, a future professional career (Wylleman et al., 2011). For elite adolescent athletes, these developmental demands are further intensified by the realities of contemporary youth high-performance sport, where frequent competition, compressed recovery windows, and substantial academic responsibilities converge within the same developmental period (Hallmann and Weustenfeld, 2025; Lundqvist et al., 2023). As a result, many young athletes must navigate a dual-career environment in which training, travel, competition, and schooling place simultaneous claims on their physiological, cognitive, and emotional resources (Hallmann and Weustenfeld, 2025; Lundqvist et al., 2023). When these demands are not effectively managed, the consequences may extend beyond transient fatigue to include attentional disruption, impaired decision-making, emotional exhaustion, sleep disturbance, and, over time, more persistent mental health difficulties and heightened risk of sport dropout (Brenner et al., 2019, 2024; Rice et al., 2016).

Crucially, this challenge is amplified by a developmental vulnerability unique to adolescence. Neurodevelopmental research has consistently described adolescence as a period of “developmental mismatch,” in which limbic systems supporting emotional reactivity and reward sensitivity become highly responsive relatively early, whereas prefrontal systems responsible for cognitive control and emotion regulation continue to mature into the mid-twenties (Ahmed et al., 2015; Shulman et al., 2016). In applied sport settings, this mismatch means that adolescent athletes may experience defeat, social evaluation, deselection, and performance pressure with near-adult emotional intensity while relying on regulatory capacities that remain under construction. Functional neuroimaging findings further suggest that adolescents often show stronger amygdala responding and less efficient prefrontal engagement during emotion regulation tasks than adults, pointing to a practical gap between emotional load and available regulatory capacity (Silvers et al., 2015; Stephanou et al., 2016). We refer to this discrepancy as a regulation gap, and we argue that it becomes especially consequential in the hours following competition, when heightened emotional activation can fuel rumination, delay downregulation, and interfere with sleep initiation and psychological recovery.

Current psychosocial support systems are not always well calibrated to this reality. Traditional psychological skills training (PST), including strategies such as cognitive reappraisal, reflective journaling, and imagery, has made an important contribution to athlete development and continues to be widely discussed in sport psychology studies (Lange-Smith et al., 2023; Park and Jeon, 2023). However, many of the PST strategies rely heavily on sustained top-down control, metacognitive effort, and access to trained practitioners; these are often the resources that are least available in the acute post-competition window, particularly for adolescents operating under fatigue, distress, and time pressure (Fuster et al., 2021; Sun et al., 2024; Sweller et al., 2019). Under these conditions, interventions that are theoretically sound in low-stress settings may become difficult to initiate, cognitively burdensome, or poorly matched to the athlete’s immediate regulatory state (Sweller et al., 2019). Compounding this challenge, help-seeking for mental health support among young and elite athletes remains constrained by stigma, access barriers, and the limited availability of specialized sport psychology services in many programs (Cosh et al., 2024). Thus, the problem is not that traditional PST is inherently flawed, but that existing support structures often fail to deliver the right type of regulation support at the precise moment it is most needed.

In this context, technology may offer a useful shift in emphasis. Rather than treating digital tools solely as instruments of monitoring, we propose that technologies can function as forms of external scaffolding for psychological recovery. Building on the concept of scaffolding originally proposed by Wood et al. (1976), such tools may temporarily assist adolescent athletes in performing regulatory functions that they cannot yet execute consistently or efficiently on their own. Importantly, our argument is not that technology could replace coaches, psychologists, or established mental skills training, but that it may serve as a complementary and bounded regulatory aid, helping athletes achieve better recovery. Accordingly, this perspective proposes a Technology-Scaffolded Regulation Framework for psychological recovery in elite adolescent athletes. The framework conceptualizes recovery as a hierarchical progression from physiological anchoring, to emotional reframing and to cognitive recharging. Our central premise is that post-competition support should be matched to the athlete’s regulatory readiness, with external scaffolds deployed strategically when self-regulation is most fragile.

## 2 Theoretical framework: the technology-scaffolded regulation model

The proposed framework rests on a theoretically important premise: psychological recovery following intense competition is not a unitary event, but a hierarchically organized process involving partially distinct physiological, emotional-regulatory and cognition systems that unfold across different time scales (Kellmann et al., 2018). We therefore argue that post-competition recovery should be conceptualized not simply as “rest,” but as a staged regulatory process in which lower-order physiological stabilization creates the conditions for subsequent cognitive and emotional recovery.

This premise is especially relevant in adolescence. Developmental models of emotion regulation suggest that adolescence is marked by a prolonged asymmetry between relatively reactive subcortical-limbic systems and still-developing prefrontal systems involved in inhibitory control, cognitive reframing, and deliberate emotion regulation (Ahmed et al., 2015; Shulman et al., 2016). In applied sport contexts, this means that young athletes may experience defeat, selection pressure, public evaluation, and disrupted routines with considerable emotional intensity while having inconsistent access to the regulatory resources required to downregulate those responses efficiently. This developmental asymmetry becomes particularly consequential in the acute hours following competition, when fatigue, arousal, disappointment, and anticipatory worry can amplify rumination and interfere with sleep onset (Juliff et al., 2015; Sim et al., 2023).

A useful psychophysiological lens for understanding the first stage of this sequence is provided by the neurovisceral integration (NVI) model (Thayer and Lane, 2000, 2009). In broad terms, NVI links adaptive self-regulation to interactions between central regulatory networks and peripheral autonomic processes indexed in part by vagally mediated heart rate variability (HRV). Within this view, HRV can be understood as a marker associated with regulatory flexibility, attentional control, and emotion regulation capacity (Thayer and Lane, 2000, 2009). Applied to the post-competition context, the model helps explain why autonomic stabilization may be an important precondition for more effortful forms of regulation.

This point is highly relevant in elite adolescent sport. In the immediate aftermath of high-stakes competition, athletes often show a profile characterized by persistent sympathetic activation, intrusive performance-related cognition, and reduced executive bandwidth, making effortful top-down strategies difficult to deploy effectively (Fuster et al., 2021; Sweller et al., 2019). From this perspective, interventions that first target breathing, arousal settling, and physiological downregulation are not merely simpler alternatives to psychological recovery; they are the functional preconditions that make subsequent forms of recovery more feasible.

Importantly, this hierarchy should not be read as a strictly one-way sequence. Consistent with Neurovisceral Integration, physiological and cognitive-emotional regulation interact bidirectionally: bottom-up autonomic settling can enable subsequent top-down regulation, while reduced rumination and cognitive settling can further support physiological recovery (Thayer and Lane, 2000, 2009). Despite this bidirectional circuitry at the mechanistic level, the immediate post-competition window imposes a practical constraint: cognitive resources and executive bandwidth are substantially depleted, such that top-down strategies cannot be reliably deployed until autonomic arousal has first been dampened. The hierarchy thus provide a practical ordering principle under post-competition constraints, rather than a rigid linear model of isolated processes.

This logic is also consistent with Conservation of Resources (COR) theory (Hobfoll, 1989), which helps explain why recovery support should be sensitive to the athlete’s depleted state. In the post-competition window, adolescent athletes may experience simultaneous strain across physiological, cognitive, and emotional domains. Interventions that demand substantial introspection, sustained concentration, or complex meaning-making at this point may therefore function as additional resource expenditure rather than recovery support (Balk and Englert, 2020). COR theory thus helps justify a sequential recovery logic, from reducing immediate strain, to supporting manageable forms of emotional processing, to facilitating later cognitive settling and restoration. Within this sequence, emotional reframing is positioned before cognitive recharging not because it is more effortful, but because unresolved disappointment, self-evaluation, and anticipatory worry may remain especially salient once acute physiological arousal has begun to subside, and may continue to sustain rumination if left unaddressed. In this sense, bounded and developmentally appropriate forms of emotional processing may help reduce the affective load that would otherwise continue to disrupt later attentional disengagement and pre-sleep recovery (Balk and Englert, 2020; Jekauc et al., 2021).

Based on these ideas, we define the present model as a Technology-Scaffolded Regulation Framework. In this formulation, technology is not conceptualized primarily as a monitoring tool, nor as a substitute for human care, but as a form of temporary external regulation that can support regulatory functioning beyond the athlete’s current unaided capacity. The scaffold is therefore compensatory rather than curative, and transitional rather than permanent. It is intended to reduce the immediate mismatch between situational demands and available self-regulatory capacity while progressively supporting internalization over time (Wood et al., 1976).

The logic clarifies why the current framework is structured into three layers. The first layer, physiological anchoring, targets autonomic settling and aims to reduce acute arousal and restore basic regulatory stability. The second layer, emotional reframing, targets the meaning-making dimension of recovery and supports bounded processing of disappointment, self-evaluation, and anticipatory worry once basic physiological stability has been restored. The third layer, cognitive recharging, targets attentional fatigue and seeks to restore directed attention, reduce residual mental activation, and support pre-sleep settling. In this sense, the hierarchy is not merely a convenient presentation device; it reflects a developmentally aligned ordering principle for intervention that matches support to the athlete’s current regulatory capacity and maturational stage (Ahmed et al., 2015; Sweller et al., 2019).

## 3 The intervention matrix: from biological substrate to psychological meaning

**Figure 1:**
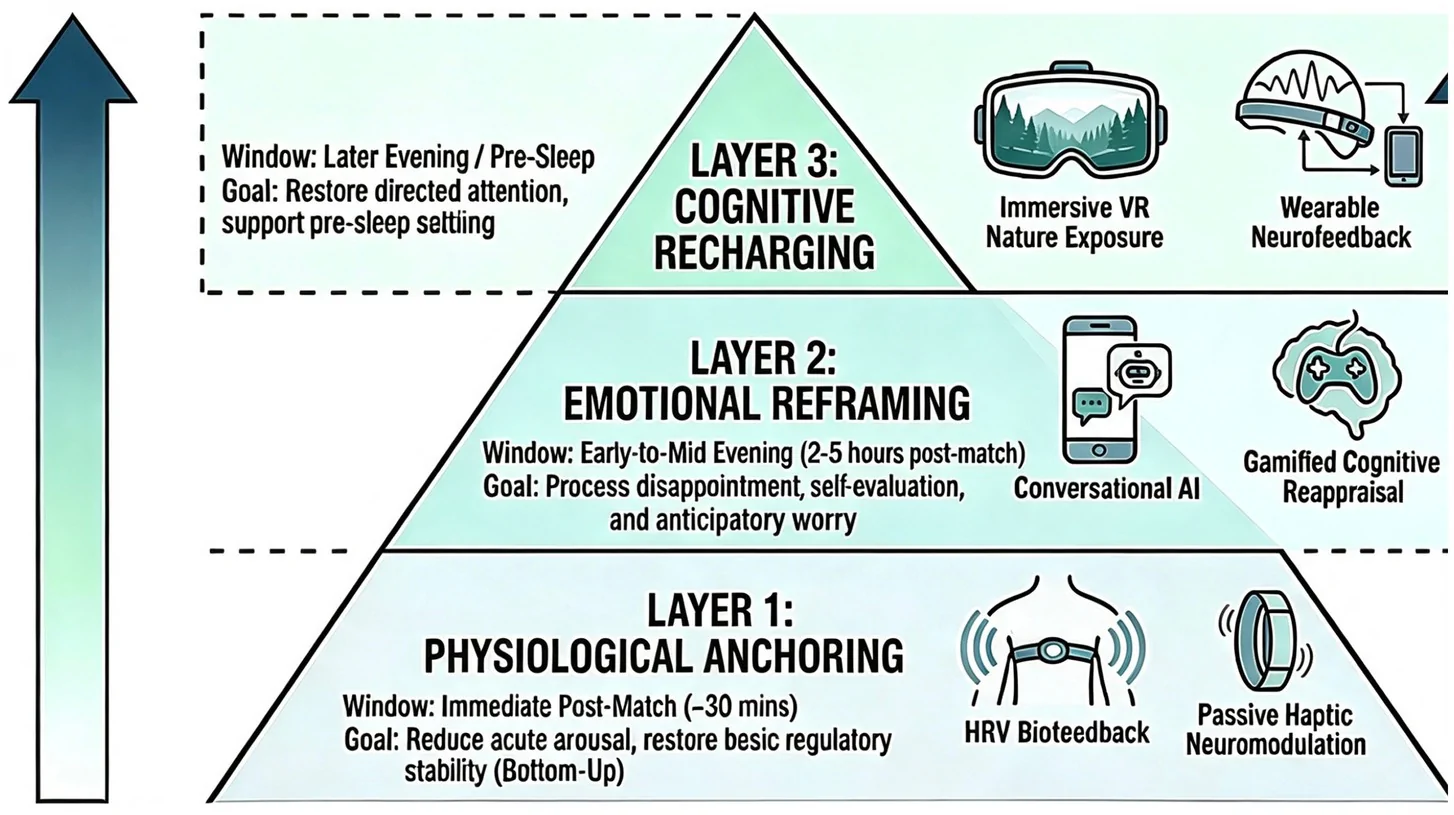
The three-layer framework.

This section translates the Technology-Scaffolded Regulation Framework into a practice-oriented intervention matrix. Rather than organizing technologies by device category, we organize them by regulatory function and temporal readiness: what the athlete is most likely able to engage with at a given point in the recovery window. The underlying premise is that post-competition interventions should match the athlete’s immediate regulatory capacity, beginning with approaches that reduce autonomic arousal before moving toward more effortful forms of reflective processing. Accordingly, the matrix is structured into three layers: physiological anchoring, emotional reframing, and cognitive recharging (i.e., Figure 1). Through a review of recent literature (see Appendix in Supplementary material for searching details), we identified promising digital strategies across these layers. Our aim is to illustrate how specific tools can function as temporary regulatory scaffolds during periods when self-regulation is least reliable and most needed.

### 3.1 Layer 1: physiological anchoring

#### 3.1.1 Window: immediate post-match (~30 min)

The first layer addresses the athlete’s most immediate regulatory need: downshifting from post-competition arousal into a physiological state compatible with recovery. Following high-stakes competition, adolescent athletes often remain in a state of heightened sympathetic activation, and standard cool-down routines such as stretching or passive rest may be insufficient to restore autonomic balance (Seiler et al., 2007). At this stage, the priority is not insight, reflection, or reframing, but rapid physiological settling. For this reason, Layer 1 interventions are intentionally low-load and predominantly bottom-up, designed to reduce arousal with minimal demands on executive control (Sweller et al., 2019).

#### 3.1.2 Biofeedback-guided respiratory modulation

In the early post-match period, it is often unrealistic to expect exhausted adolescent athletes to engage immediately in effortful reflective processing. By contrast, physiologically oriented regulation centered on paced breathing may provide a more accessible entry point for recovery. As the immediate priority at this stage is to reduce acute arousal and restore basic autonomic stability, interventions that act first at the physiological level are likely to be more acceptable than strategies that depend on sustained top-down control (Ahmed et al., 2015; Sweller et al., 2019).

On this basis, HRV biofeedback is positioned in the present framework as one of the more empirically grounded options, as it directly targets the athlete’s most immediate post-competition regulatory need: autonomic settling (Dormal et al., 2021; Lehrer and Gevirtz, 2014). Specifically, HRV biofeedback uses real-time feedback to guide the athlete toward slow, regular breathing near their individual resonance frequency (RF), which typically falls between 4.5 and 6.5 breaths per minute in adults but tends to be somewhat higher in children and adolescents, around 6.5 to 9.5 breaths per minute, reflecting developmental differences in respiratory and autonomic physiology (Lehrer and Gevirtz, 2014; Shaffer and Meehan, 2020). Given this variability, RF should ideally be determined through a brief individualized assessment; where time constraints preclude this, a default rate adjusted toward the upper end of the pediatric range (i.e., approximately 7–8 breaths per minute) may be more appropriate than adult-derived defaults such as six breaths per minute, particularly given evidence that HRV biofeedback is both feasible and effective in adolescent populations (Dormal et al., 2021). Such pacing is intended to facilitate vagally mediated regulatory processes and attenuate excessive sympathetic activation. In practice, athletes may wear an HRV-monitoring device such as the Polar H10 immediately after competition and complete approximately 6 min of resonance/mindful breathing as an initial intervention within the post-match recovery window (Zhu et al., 2022).

#### 3.1.3 Passive haptic neuromodulation

Passive haptic neuromodulation may serve as a supplementary Layer 1 option for athletes who are too fatigued, restless, or cognitively overloaded to engage even in guided breathing (Fooks and Niebuhr, 2024; Hallihan and Siegle, 2022). Its proposed value lies in offering a near-effortless route to physiological settling, using patterned vibration or tactile stimulation to support downregulation without requiring sustained attention or reflective effort (Fooks and Niebuhr, 2024; Hallihan and Siegle, 2022). For example, in a pilot study of 22 university athletes, calming vibrations (89 Hz modulated by 0.1 Hz, 40 Hz modulated by 0.1 Hz, or 89 Hz modulated by 4 Hz) delivered through a wrist-worn wearable transducer during a 2-min post-exercise (i.e., acute arousal by 30s cycling) recovery period, individually calibrated to each athlete’s subjective response, were associated with increased high-frequency heart-rate variability (i.e., means enhanced parasympathetic tone), particularly among those most physiologically affected by prior exertion (Hallihan and Siegle, 2022). Complementary evidence from a full-body vibroacoustic platform delivering a 45-min nature-based soundscape with 40 Hz bass frequencies further suggests that such stimulation can increase mean HRV by nearly 30%, significantly improve parasympathetic markers, and reduce heart rate by approximately 15%, with relaxation effects persisting beyond the stimulation period (Fooks and Niebuhr, 2024). As these studies differ considerably in delivery modality (localized vs. full-body), frequency, and dosage (2 vs. 45 min), the evidence base for passive haptic approaches, while promising, remains preliminary, and future comparative trials are needed.”

### 3.2 Layer 2: emotional reframing

#### 3.2.1 Window: early-to-mid evening (approx. 2–5 h post-competition)

The second layer addresses the meaning-making dimension of recovery, which often becomes more salient once acute physiological arousal has begun to subside but disappointment, self-evaluation, and anticipatory worry remain active (Balk and Englert, 2020; Jekauc et al., 2021). At this stage, the goal is not only to reduce residual arousal or fatigue but also to support the athlete in processing disappointment, self-evaluation, and anticipatory worry in ways that reduce rumination and prepare the ground for subsequent adaptation (Balk and Englert, 2020; Jekauc et al., 2021). As such work places greater demands on reflective capacity, Layer 2 interventions are introduced only once more basic physiological regulation has been at least partially restored.

#### 3.2.2 Conversational AI as a co-regulation partner

Conversational AI may serve as a supplementary Layer 2 tool for athletes who remain emotionally activated after competition but are not ready to engage in more effortful or socially demanding forms of support. Its proposed value lies in offering an immediate and relatively low-pressure format for brief emotional processing when formal psychological support is unavailable. This positioning is broadly consistent with evidence that AI-driven conversational agents can reduce depressive symptoms among young people, although direct evidence in elite adolescent post-competition settings remains limited (Feng et al., 2025). Drawing on principles from cognitive-behavioural approaches and process models of emotion regulation (Beck and Haigh, 2014; Gross, 2015), a short post-competition interaction may be structured around four steps: (1) emotion labeling; (2) brief validation without evaluative judgment; (3) cognitive distancing or perspective-taking; and (4) a concrete, recovery-oriented next action. The purpose is not to analyse the match, determine causes of performance, or provide psychotherapy, but to help the athlete name the immediate affective state and shift attention toward a manageable recovery action. To reduce the risk of rumination, the interaction should remain brief, avoid repeated probing of disappointment or self-blame, and defer detailed performance analysis to a later coach- or psychologist-led debrief.

As the relational chatbot styles may be especially appealing to adolescents, particularly those who are socially or emotionally vulnerable, any deployment within this framework should prioritise a transparent, non-relational framing and clearly limit the agent’s non-diagnostic and non-therapeutic role (Kim et al., 2025). Given limited reporting of ethical and safety standards in the youth conversational-agent literature (Hawke et al., 2025), we recommend that implementation include, at minimum: real-time escalation when markers of acute distress emerge; explicit communication that the agent is not a mental-health professional; and a predefined referral pathway to a qualified sport psychologist or mental health professional when clinically significant distress, self-harm risk, or safeguarding concerns are indicated. Concerns regarding privacy, data security, and algorithmic bias also remain substantial for this population (Tao, 2026; Tao et al., 2026; Wu et al., 2026). For these reasons, conversational AI is best positioned not as a standalone recovery tool, but as a tightly bound, transparently framed adjunct for short-form emotional debriefing alongside trusted human support.

#### 3.2.3 Gamified cognitive reappraisal

Gamified cognitive reappraisal may serve as a useful Layer 2 tool by making reflective emotional processing more accessible to adolescents under conditions of fatigue and reduced motivation. Rather than requiring abstract journaling (e.g., CBT-style exercises) or effortful self-analysis, gamified formats can present reframing through structured prompts, narrative elements, and reward-based progress cues. In current framework, cognitive reappraisal remains the active regulatory mechanism supported by narrative reframing and emotional distancing, and gamification serves primarily to lower the effort required to engage with reappraisal when athletes are fatigued. Following Cheng and Ebrahimi’s (2023) three-stage account, motivational affordances are expected to foster engagement and compliance, which may then support psychological change processes within the intervention setting and, ultimately, more adaptive recovery-related outcomes. This delivery logic is consistent with emerging work in performance sports in which gamified elements such as challenges, rewards, and progress tracking have been used to support athlete engagement and adherence in rehabilitation and recovery-related routines (Biró and Szilágyi, 2025). Tools such as Mightier illustrate this design principle but are not proposed as validated elite-sport protocols (Mannweiler et al., 2023). Evidence from youth mental health and school-based settings further suggests that gamified interventions can support emotion regulation and coping, although direct evidence in elite adolescents during the acute post-competition window remains limited (Cheng and Ebrahimi, 2023; David et al., 2024; Grunewald et al., 2025; Reynard et al., 2022).

### 3.3 Layer 3: cognitive recharging

#### 3.3.1 Window: later evening/pre-sleep

Once acute physiological arousal has begun to settle, the next challenge is often cognitive depletion. Tournament blocks, travel, repeated decision-making, and performance monitoring can drain directed attention, leaving athletes mentally “switched on” but unable to concentrate effectively or disengage from performance-related thoughts (Habay et al., 2021; Sun et al., 2024). Layer 3 is therefore focused on restoring attentional resources by minimizing cognitive demands rather than adding new ones (Kaplan, 1995; Ohly et al., 2016). The interventions in this layer are intended to create conditions in which the fatigued attentional system can recover, thereby reducing residual mental activation and supporting the transition toward sleep.

#### 3.3.2 Immersive nature exposure via virtual reality

Immersive nature exposure via virtual reality may serve as a useful tool because it targets attentional fatigue without imposing substantial additional cognitive demands. By providing a form of simulated “soft fascination,” VR nature environments may allow the directed attention system to rest and recover (Browning et al., 2020; Mattila et al., 2020), even when people are confined to cognitively sterile or stressful settings (Li et al., 2021; Ohly et al., 2016). Emerging evidence suggests that virtual nature exposure can improve perceived restoration and aspects of cognitive functioning, although most studies have been conducted in adult or non-athlete samples rather than elite adolescent sport contexts (Browning et al., 2020; Han et al., 2025; Li et al., 2021; Mattila et al., 2020). In sport context, Sun et al. (2022) demonstrated that at least 12.5 min of nature exposure can alleviate mental fatigue and significantly enhance decision-making speed and accuracy among university soccer players. Furthermore, recent evidence indicates that exercising in a nature-based VR environment reduces cortisol and perceived exertion while increasing enjoyment, with a near-significant trend toward elevated *β*-endorphin release (Babaie et al., 2026). Within the present framework, VR is therefore positioned as a cognitively light recharging tool that may help restore attentional resources and support the transition toward pre-sleep settling.

#### 3.3.3 Wearable neurofeedback for pre-sleep settling

Wearable neurofeedback offers a low-burden, state-modulating strategy to facilitate pre-sleep downregulation in athletes who remain cognitively activated by performance-related thoughts, a common situational driver of post-competition sleep disturbance (Juliff, 2017; Juliff et al., 2015). Unlike conventional neurofeedback framed as effortful cognitive training, portable implementations externalize monitoring through simple auditory or multisensory cues, thereby reducing reliance on endogenous attentional control during periods of depleted cognitive capacity. In practice, the intervention is envisaged as a portable dry-electrode EEG wearable (e.g., a lightweight headband), typically targeting alpha activity (8–12 Hz) with frontal or sensorimotor montages; alpha is prioritized because it is commonly linked to reduced cortical arousal and cognitive stillness, while alpha–theta protocols may be considered as an alternative for relaxation and sleep facilitation (Recio-Rodriguez et al., 2024). Feedback is preferably auditory (usable with eyes closed), with optional vibrotactile cues when needed, whereas continuous visual displays are generally less suitable immediately before sleep. The athlete’s role is limited to low-effort state modulation rather than active strategy use, typically by sitting or lying quietly and attending lightly to the feedback.

In sport context, current reviews indicate that neurofeedback could accelerate recovery, enhance reflexes and coordination, improve attention and emotional regulation, and, across a range of sports and competitive levels, produce overall improvements in athletic and cognitive performance as well as anxiety management (Rydzik et al., 2023; Tosti et al., 2024). Additionally, Semertzidis et al. (2019) reported that, in a non-sport sample, participants showed significantly decreased pre-sleep cognitive arousal, negative emotion, and negative affect, alongside EEG indices consistent with more restorative pre-sleep states. Collectively, these findings support the potential of wearable neurofeedback as an unobtrusive adjunct for mitigating pre-sleep rumination and over-arousal in mentally fatigued athletes, while underscoring the need for larger, well-controlled trials to define optimal parameters.

## 4 Implementation and ethical considerations

Implementation must remain pragmatically flexible. Under congested schedules or following late-evening competition where recovery windows are severely truncated, practitioners may need to adapt the hierarchy, such as bypassing Layer 2 emotional reframing to prioritize rapid physiological anchoring (Layer 1) and pre-sleep cognitive recharging (Layer 3) to protect sleep architecture.

Successful implementation requires clear role allocation and gradual skill familiarization. Sport psychologists or mental performance consultants should oversee selection, onboarding, and supervision; coaches should support routine integration within ordinary training recovery; and, when specialist support is limited, athletes should first practise the tools in low-stakes training contexts so that procedural familiarity is established and the selected intervention can be shown to be reasonably effective before use in emotionally demanding post-competition settings.

Ethically, the use of these tools needs to remain bounded by attention to athlete readiness, privacy, and appropriate human oversight. Given the developmental vulnerabilities of adolescents, deploying tools like conversational AI necessitates robust safeguards against data privacy risks, algorithmic biases, and the potential for digital over-reliance (Tao et al., 2026). Ultimately, because these tools are temporary, developmentally sensitive scaffolds rather than substitutes for human care, their success depends on being embedded within the broader support ecology and progressively faded as the young athlete’s internal regulatory capacities mature.

## 5 Conclusion

Elite adolescent athletes are often required to manage post-competition demands that exceed their immediately available self-regulatory resources, particularly under conditions of cumulative fatigue, dual-career pressure, and compressed recovery windows. This perspective argues that technology can be understood not only as a monitoring system, but also as a form of temporary external scaffolding that helps bridge this gap in developmentally appropriate ways. By organizing recovery hierarchically, from physiological anchoring to cognitive recharging, the current framework provides a concise implementation pathway for aligning post-competition support with adolescent athletes’ psychological recovery.

## Data Availability

The original contributions presented in the study are included in the article/Supplementary material, further inquiries can be directed to the corresponding author.
