# Supplementary material for: Post-competition psychological recovery in elite adolescent athletes: a technology-scaffolded perspective
Source: Front Psychol. 2026 Aug 13;17:1902066. doi: 10.3389/fpsyg.2026.1902066 (PMC13518143; doi:10.3389/fpsyg.2026.1902066)
Supplement: Supplementary file 1 [file Supplementary_file_1.DOCX]

**Appendix**

**Literature search strategy**:

A structured search was conducted to ensure the proposed framework was informed by the most current and relevant empirical evidence. The literature search was conducted in Dec/2025 across major electronic databases, including Web of Science and EBSCOhost (including PsycINFO, CINAHL, and SPORTDiscus). The retrieved findings were selectively synthesized to provide the conceptual and empirical foundation for the arguments and intervention strategies presented throughout this study.

**Overall Framework**

**String**:

("young athlete*" OR "adolescent athlete*" OR "youth athlete*" OR "junior athlete*") AND ("digital" OR "mobile" OR "app" OR "wearable" OR "VR" OR "virtual reality" OR "biofeedback" OR "HRV" OR "gamif*" OR "serious game*" OR "chatbot" OR "AI") AND ("mental*" OR "psychological recovery" OR "emotion regulation" OR "stress recovery" OR "anxiety" OR "post-match" OR "between-match" OR "tournament")

**Web of Science**:

Limited to the past 10 years.

Results yielded: 38.

**EBSCOhost**:

Limited to the past 10 years and peer-reviewed articles.

Results yielded: 7.

**Layer 1: Physiological Anchoring**

**String**:

("adolescent*" OR "youth" OR "young athlete*" OR "junior athlete*") AND ("heart rate variability" OR HRV OR "respiratory sinus arrhythmia" OR RSA OR "vagal tone" OR "parasympathetic" OR "autonomic balance" OR biofeedback OR neurofeedback OR "haptic feedback" OR vibrotactile OR "affective touch" OR "resonant breathing" OR "slow breathing" OR "coherent breathing" OR "wearable*" OR "Apollo Neuro" OR "TouchPoints" OR "Sensate") AND (stress OR anxiety OR recovery OR arousal OR "physiological recovery" OR "post-exercise recovery" OR "post-match" OR fatigue) AND (trial OR randomized OR RCT OR feasibility OR pilot OR review)

**Web of Science**:

Limited to the past 10 years and the English language.

Results yielded: 373.

**EBSCOhost**:

Limited to the past 10 years.

Results yielded: 92.

**Layer 2: Emotional Reframing**

String: ("adolescent*" OR "youth" OR "young athlete*" OR "junior athlete*") AND (gamif* OR "serious game*" OR "digital game-based" OR "applied game*") OR (chatbot* OR "conversational agent*" OR "dialogue system*" OR "AI companion" OR "artificial intelligence" OR "generative AI" OR "large language model") AND ("emotion regulation" OR "cognitive reappraisal" OR "emotional processing" OR "post-event processing" OR rumination OR "psychological debrief*" OR "mental recovery" OR "affective recovery" OR mood OR anxiety OR stress) AND (trial OR randomized OR feasibility OR pilot OR acceptability OR engagement OR adherence OR review)

**Web of Science**:

Initial limits included the past 10 years and English language (yielding 3,637 results). Due to the extensive volume of existing evidence, the authors applied a secondary filter to prioritize the most contemporary synthesized evidence. The search was further limited to review articles published between 2024 and 2025. Final results retained: 808.

**EBSCOhost**:

Limited to the past 10 years.

Results yielded: 184.

**Layer 3: Cognitive Recharging**

**String**:

("adolescent*" OR "youth" OR "young athlete*") AND ("virtual reality" OR VR OR "360 video" OR "virtual nature" OR "nature exposure" OR "restorative environment*" OR "attention restoration" OR "directed attention" OR "mental fatigue" OR "cognitive fatigue" OR "decision fatigue" OR "ego depletion") AND (recovery OR restoration OR "cognitive recovery" OR "mental recovery" OR stress OR fatigue) AND (trial OR randomized OR experiment* OR RCT OR "systematic review")

**Web of Science**:

Limited to the past 10 years and English language.

Results yielded: 119.

**EBSCOhost**:

Limited to the past 10 years.

Results yielded: 31.
